# Fabricating Precise and Smooth Microgroove Structures on Zr-Based Metallic Glass Using Jet-ECM

**DOI:** 10.3390/mi15040497

**Published:** 2024-04-04

**Authors:** Dongdong Li, Pingmei Ming, Shen Niu, Guangbin Yang, Kuaile Cheng

**Affiliations:** School of Mechanical and Power Engineering, Henan Polytechnic University, Jiaozuo 454003, China; li863964931@163.com (D.L.); ns2019@hpu.edu.cn (S.N.);

**Keywords:** Zr-based metallic glasses (MGs), jet-ECM, precise and smooth microgroove structures, sodium nitrate electrolyte

## Abstract

Zr-based metallic glasses (MGs) are promising materials for mold manufacturing due to their unique mechanical and chemical properties. However, the high hardness of metallic glasses and their tendency to crystallize at high temperatures make it challenging to fabricate precise and smooth microscale structures on metallic glasses. This limitation hampers the development of metallic glasses as molds. Jet electrochemical machining (jet-ECM) is a non-contact subtractive manufacturing technology that utilizes a high-speed electrolyte to partially remove material from workpieces, making it highly suitable for processing difficult-to-machine materials. Nevertheless, few studies have explored microgroove structures on Zr-based MGs using sodium nitrate electrolytes by jet-ECM. Therefore, this paper advocates the utilization of the jet-ECM technique to fabricate precise and smooth microgroove structures using a sodium nitrate electrolyte. The electrochemical characteristics were studied in sodium nitrate solution. Then, the effects of the applied voltages and nozzle travel rates on machining performance were investigated. Finally, micro-helical and micro-S structures with high geometric dimensional consistency and low surface roughness were successfully fabricated, with widths and depths measuring 433.7 ± 2.4 µm and 101.4 ± 1.6 µm, respectively. Their surface roughness was determined to be 0.118 ± 0.002 µm. Compared to non-aqueous-based methods for jet-ECM of Zr-based MGs, the depth of the microgrooves was increased from 20 μm to 101 μm. Furthermore, the processed microstructures had no uneven edges in the peripheral areas and no visible flow marks on the bottom.

## 1. Introduction

The dependable large-scale manufacturing of components with micro-/nanoscale features is crucial for the rapid advancement of microelectromechanical systems (MEMS) [1]. Polymers are the most commonly used substrate materials in microelectromechanical systems (MEMS) due to their low cost, chemical corrosion resistance, and the availability of fabrication processes [2]. Polymer surface microstructures are obtained by replicating mold structures. Currently, a variety of materials are employed as molds, including electroformed nickel [3], silicon [4], and stainless steel [5]. However, these materials have the disadvantages of poor precision and short service life. Metallic glasses have outstanding mechanical properties, such as high strength and corrosion resistance, which allow them to be used as mold materials [6,7,8]. Nevertheless, the fabrication of surface microstructures on metallic glass molds presents a significant challenge. 

At present, thermoplastic forming has been demonstrated as a prevalent technique for microstructures based on metallic glasses [9]. However, the method requires precise control of temperature and duration to prevent crystallization and oxidation [10]. Micro-subtractive manufacturing exhibits a clear advantage in terms of processing precision and repeatability for fabricating microstructures in metallic glasses. This primarily includes techniques such as cutting, electrical discharge machining, laser processing, etc. [11,12,13,14]. In the cutting process, the high hardness and toughness of amorphous alloys can lead to significant tool wear, consequently diminishing the operational lifespan of the tool [15]. In addition, electrical discharge machining and laser machining are regarded as thermal processing techniques. During the machining process, certain defects may arise, such as recast layers, heat-affected zones, and microcracking [16,17,18]. 

Electrochemical machining (ECM) removes material through anodic dissolution via electrochemical reactions without generating cutting forces or heat-affected zones during the machining process. Koza et al. [19] employed microtool electrodes for the electrochemical machining of Zr-based bulk metallic glass and confirmed that NaNO_3_ aqueous solution is not suitable for electrochemical machining of Zr-based MG due to the formation of corrosion products. Furthermore, they found that using a methanol–HClO_4_ solution could reduce the precipitation of corrosion products. However, methanol–HClO_4_ solutions cannot be widely used due to their toxicity. Similarly, Geber et al. [20] asserted the impracticality of using standard water-based salt electrolytes for the electrochemical machining of Zr-based metallic glass. Cole et al. [21] discovered that the corrosion products and the dense oxides generated in water-based electrolytes hindered the electrochemical machining (ECM) of Zr-based MG. To address this issue, they adopted a method of increasing the bias voltage to reduce the adhesion of corrosion products to the machined surface. Subsequently, Guo et al. [22,23,24] proposed the utilization of non-aqueous-based methods for pulse electrochemical machining of microstructures on Zr-based bulk metallic glass. However, the machined microstructures exhibited uneven edges in the peripheral regions and pronounced flow marks on the bottom. Additionally, electrochemical machining of Zr-Based MGs using organic solvent electrolytes results in low material removal rates, limiting fabrication to shallow microstructures. In the past decade, Zeng et al. [25,26,27] proposed the utilization of WECMM technology to manufacture high-precision and -quality microstructures in metallic glasses using water-based electrolytes. However, wire electrochemical micromachining faces difficulties in machining blind holes, grooves, and cavities, as well as three-dimensional complex structures and parts.

Jet electrochemical machining is a technology that uses a hollow metal nozzle as the cathode, enabling the electrolyte to be sprayed directly onto the workpiece surface from within the metal nozzle. This technology utilizes the electrochemical anodic dissolution principle to achieve material removal from specific locations on the workpiece [28]. In comparison to alternative ECM techniques, jet-ECM employs high-speed electrolyte flushing to address the adhesion issues of electrolytic products encountered in electrochemical machining. Jet-ECM has shown significant promise as a shaping method. Hackert-Oschatzchen et al. [29] fabricated complex microstructures on stainless steel surfaces using tubular electrodes with an inner diameter of 0.1 mm to mill microgrooves measuring 200 μm in width and 60 μm in depth. Liu et al. [30] investigated the influence of process parameters on the surface groove structure of a titanium alloy through jet electrochemical machining. Ultimately, they utilized optimal process parameters to fabricate an S-shaped groove structure.

To the best of our knowledge, there have been no reports on the study of precise and smooth microgroove structures on Zr-based metallic glass produced using sodium nitrate electrolytes by jet electrochemical machining thus far. Therefore, this paper assesses the feasibility of jet electrochemical machining for fabricating precise, smooth microgroove structures on Zr-based MG. Firstly, the electrochemical characteristics of the Zr-based MG in NaNO_3_ solution are discussed. Then, the effects of the applied voltage and the nozzle travel rate on the machining quality of microgrooves on the Zr-based MG were investigated. Finally, micro-helical and micro-S structures with high geometric consistency and low surface roughness were successfully fabricated, featuring widths and depths of 433.7 ± 2.4 µm and 101.4 ± 1.6 µm, respectively. Their surface roughness measures were 0.118 ± 0.002 µm.

## 2. Materials and Methods

### 2.1. Sample Preparation

The Zr-based metallic glass (MG) was obtained through copper mold suction casting. The Zr-based metallic glass (Zr_41.2_Ti_13.8_Cu_12.5_Ni_10.0_Be_22.5_) is named Vit1. The major metal component is Zr, with a mass fraction of 41.2%, and the secondary metal elements are Be and Ti, with mass fractions of 22.5% and 13.8%, respectively. Additionally, 10.0% nickel and 12.5% copper were added to the Zr-based MG. The material properties of the Zr-based MG are presented in Table 1. The workpiece materials (20 mm (W) × 20 mm (L) × 2 mm (T)) were used in jet-ECM. The surfaces were polished with waterproof abrasive paper and ultrasonically cleaned with deionized water and ethanol before the experiments.

### 2.2. Electrochemical Measurement Setup

**Figure 1:**
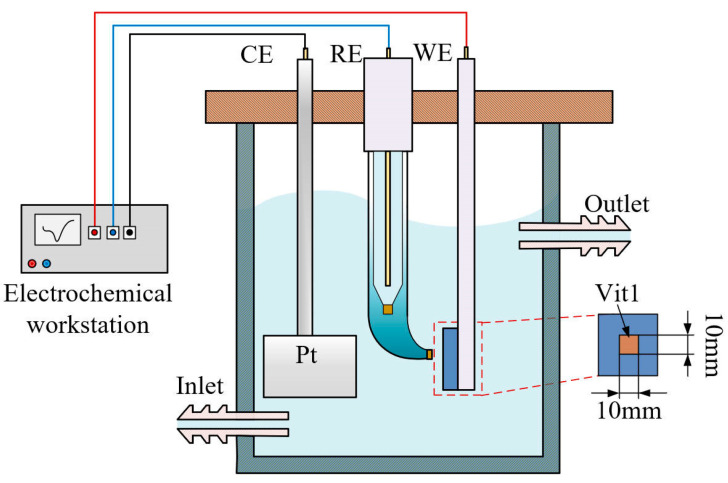
Electrochemical measurement setup.

The electrochemical characteristics were measured in a three-electrode system using an electrochemical workstation (CHI604E, CH Instruments, Shanghai, China), as shown in Figure 1. A platinum plate was used as a counter electrode (CE). The reference electrode (RE) employed was Hg/Hg_2_Cl_2_, which was in contact with the electrolyte via a salt bridge to reduce the liquid junction potential. The Vit1 (10 mm (W) × 10 mm (L) × 2 mm (T)) was insulated with epoxy resin as the working electrode (WE), and only 1 cm^2^ of surface area was exposed to the electrolyte. The open circuit potential (OCP) was monitored in NaNO_3_ solution until a stable surface state was reached before the electrochemical measurements.

Polarization tests were conducted with a scan rate of 10 mV s^−1^. The potential range was −1 V to 3 V vs. Hg/Hg_2_Cl_2_. To obtain details of the interface structure of the passive film on the Vit1 in NaNO_3_ solution under different corrosion states, electrochemical impedance spectroscopy (EIS) was employed over a frequency range of 100 kHz to 0.1 Hz with a disturbance amplitude set to 5 mV. The EIS fitting data for the Zr-based MG were determined using ZView2 software.

### 2.3. Experimental System for Jet-ECM

**Figure 2:**
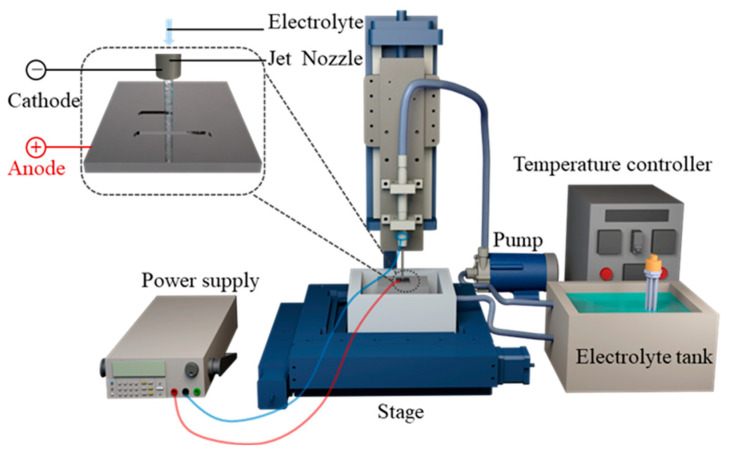
The experimental setup for processing the Vit1 by jet-ECM.

The experimental setup was conducted in NaNO_3_ solutions using a homemade experimental system, as depicted in Figure 2. The system included a 3D XYZ stage, a power supply, an electrolyte circulation unit, and a motion control unit. The anode workpiece was the Vit1, and a hollow SUS304 nozzle was chosen as the cathode. The hollow nozzle and anode workpiece were installed on the Z-stage and XY-stage, respectively. The electrolyte was ejected from the nozzle at a relatively constant high speed by a pump. The positioning and movement operations between the anode and the cathodic substrate were precisely controlled by a computer numerical control system. A groove was machined on the surface of the Vit1 through electrochemical milling. All experiments were carried out under the conditions listed in Table 2.

### 2.4. Test Equipment

A field emission scanning electron microscope (Merlin Compact, Carl Zeiss NTS GmbH, Jena, Germany) was used to analyze the topography of the sample. The surface morphology and the surface roughness were measured by a confocal laser scanning microscope (OLS5100, Olympus, Tokyo, Japan). A photograph of the microstructure as obtained using a camera (Z5, Nikon, Tokyo, Japan).

**Figure 3:**
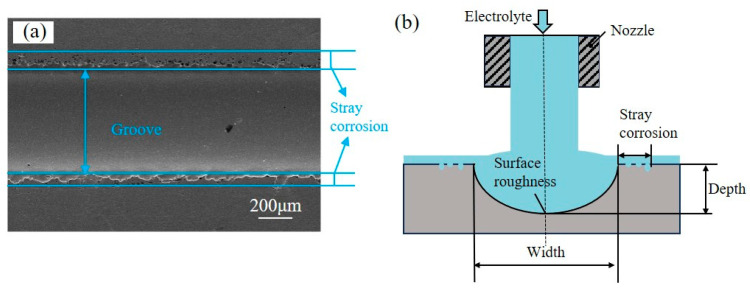
Profile of the machined groove: (**a**) SEM picture of the detailed stray corrosion; (**b**) schematic of performance evaluation indices.

The performance evaluation criteria for machining in the experiment included width, depth, aspect ratio, surface roughness, and stray corrosion, as shown in Figure 3. The aspect ratio is defined as the ratio of depth to width. Figure 3a exhibits an SEM picture of the groove profile machined by jet-ECM with an applied voltage of 20 V and a nozzle travel rate of 100 μm/s. Stray corrosion is defined in Figure 3a. 

## 3. Results and Discussion

### 3.1. Electrochemical Characteristic Analysis of the Vit1

#### 3.1.1. Anodic Polarization Curves

**Figure 4:**
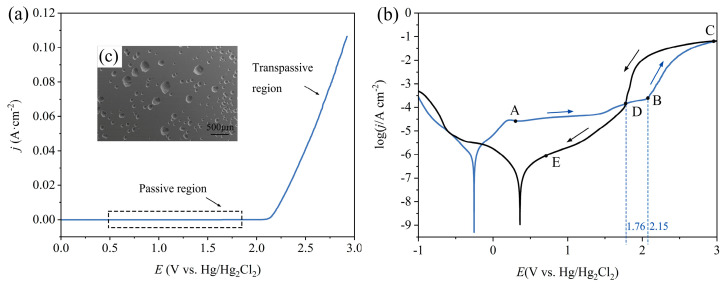
Polarization curve of the Vit1 in NaNO_3_ solution: (**a**) linear sweep voltammetry curve; (**b**) cyclic voltammetry curve; (**c**) SEM image of the transpassive region.

The anodic electrochemical characteristics of the Vit1 in a 10 wt% NaNO_3_ solution at a temperature of 25 °C were investigated after the surface stabilized. A linear sweep voltammetry (LSV) curve is shown in Figure 4a. When the voltage is below 2.15 V, the passive film remains intact, and the current density does not change with increasing potential, forming a passive region. When the voltage exceeds the breakdown potential (2.15 V), the curve exhibits an ohmic (R_oh_) behavior [31]. The current increases with voltage, which indicates that the electrochemical dissolution enters the transpassive region. At this stage, the passive film ruptures, leading to pitting corrosion. As illustrated in Figure 4c, pits are distributed on the sample surface, resulting in a uniform and rough surface morphology.

E_corr_ and E_trans_ are the corrosion potential and the transpassivation potential, respectively. As shown in Figure 4b, the corrosion potential is E_corr_ (−0.27 V), corresponding to the minimum value of the polarization curve, which is conducive to the formation of passive film in the air. When the potential exceeds −0.27 V, the corrosion current density of the Vit1 increases rapidly, reaching 31.7 μA cm^−2^ at 0.13 V. When the polarization potential is between 0.13 and 1.48 V, the current density remains nearly constant, and a passive film forms on the sample surface, hindering the anodic reaction. As the potential is increased from 1.48 V to 2.15 V, the current density increases from 50.6 to 363.5 μA cm^−2^. When the potential exceeds E_trans_ (2.15 V), pitting corrosion starts to appear on the surface of the anodic specimen. Weak points in the passivation film can be penetrated. Therefore, the resistance of the passivation film decreases, leading to an increase in current.

A reverse scan was conducted to investigate the re-passivation performance of the Vit1 in NaNO_3_ solution. Upon reaching a potential of 3 V during the reverse scan (regions C and D), the current density rapidly decreases as the voltage descends. In the pitting area, the passive film is re-established. In the phase D–E, the deceleration in the rate of current decrease is attributed to the increase in resistance. Point D in the cyclic voltammetry curve is the intersection of the forward voltage scan and the reverse voltage scan. The presence of point D indicates that the pitting generated during the forward voltage scan is filled during the reverse scan [32].

#### 3.1.2. Electrochemical Impedance Spectroscopy (EIS)

In the NaNO_3_ electrolyte, electrochemical impedance spectroscopy (EIS) tests were performed on three typical DC potentials in the polarization curve: 0.1 V (Tafel region), 1 V (passive region), and 2.1 V (near pitting onset) vs. Hg/Hg_2_Cl_2_. 

**Figure 5:**
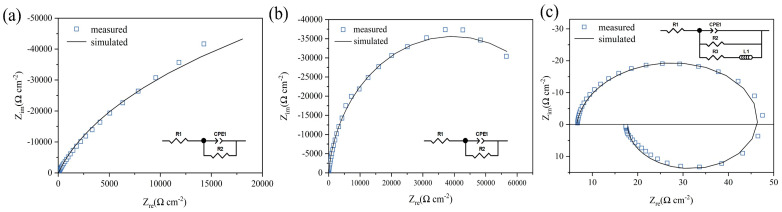
EIS results during polarization at different potentials: (**a**) 0.1 V; (**b**) 1 V; (**c**) 2.1 V.

Table 3 displays the EIS data and fitting results for the Vit1. The equivalent circuit is proposed, mainly consisting of the solution resistance (R_1_), charge transfer resistance (R_2_), constant phase element (CPE), and inductance element (L_1_). R_1_ is related to the dissolution resistance of the workpiece material. Z_CPE_ represents the capacitance of the passivation film, which is defined as follows [33]:

Z_CPE_ = [Q(jw)^n^]^−1^ (1)

where Q is the constant of the CPE, j is the imaginary number (*j*^2^ = −1), w is the angular frequency, and n is the deviation parameter, which is used to gauge surface heterogeneity. The closer n is to 1, the more uniform the surface [34,35]. The EIS data (scatter plot) and the fitting data (straight line) are shown in Figure 5. The fitted values for all the variables are listed in Table 3. At a potential of 0.1 V, the initial surface forms a monolayer of passive film, typically exhibiting a relatively dense structure. R_2_ exhibits the highest value, and the value of n_1_ is equal to 0.91, indicating the presence of pores within the typically dense internal structure of the passive film. A Nyquist plot of the polarization process at 1 V is illustrated in Figure 5b. The appearance of a semicircle is attributed to the oxidation of the base metal during the electrochemical dissolution process. As depicted in Figure 5c, increasing the polarization potential to 2.1 V leads to an evident reduction in the impedance modulus and the appearance of an inductive loop. The charge transfer resistance (R_2_) decreases to 44.18 Ω·cm^2^, indicating that the material begins to dissolve in the solution.

#### 3.1.3. Discussion of the Material Removal Region in Jet-ECM

Jet-ECM processing uses a nozzle as the cathode, and an electrolyte is sprayed out from the cathode of the nozzle. The electrolyte is dispersed radially around the center of the nozzle, forming a thin layer of electrolyte of a certain thickness around the nozzle. The distribution of the electric field determines the magnitude of the current density. Low electric field intensities and current densities are distributed in regions far from the electrode center. In the high-current-density region, the passive film ruptures or reaches a dynamic equilibrium stage of transpassivation, leading to the workpiece material being rapidly removed. The passive film is regenerated in the low-current-density region [36]. The passive film prevents the material from contacting the electrolyte and thus plays a protective role.

### 3.2. Parametric Effects of Microgroove Fabrication by Jet-ECM

#### 3.2.1. Effects of Applied Voltage

Applied voltage plays a crucial role in determining the precision, surface quality, and morphology characteristics in electrolytic processing. In order to investigate the effect of applied voltage on microstructural morphological changes and geometric contours, jet-ECM was carried out with the following processing parameters: a NaNO_3_ electrolyte concentration of 10 wt%, an initial gap of 200 μm, a nozzle travel rate of 100 μm/s, and an applied voltage of 10 to 25 V.

**Figure 6:**
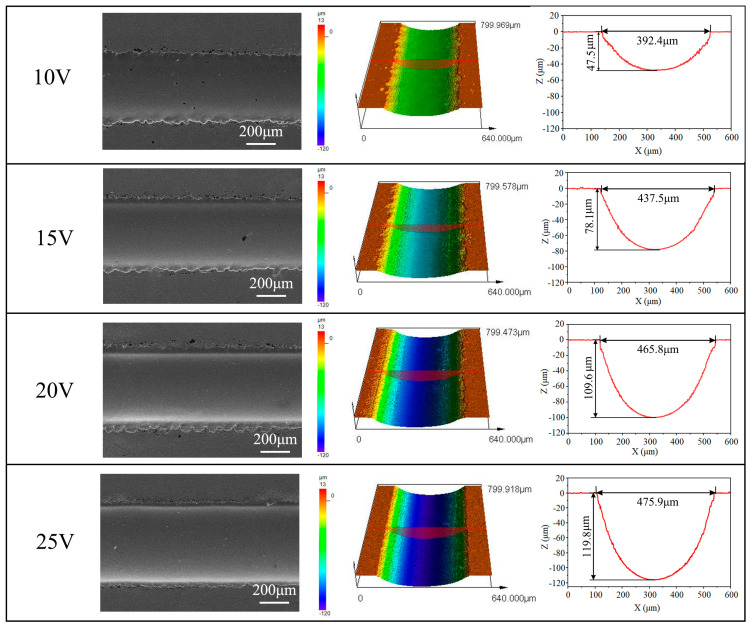
Morphology and profiles of microgrooves at different applied voltages.

Figure 6 shows the microgroove morphology and cross-sectional profiles at different applied voltages. When the voltage was 10 V and 15 V, the microgroove structure contour was not clear, the width of the microgroove was only 392.4 μm and 437.5 μm, and the depth was only 47.5 μm and 78.1 μm. When the applied voltage was increased to 20 V, the material dissolution increased and the width and depth of the microgroove were 465.8 μm and 109.6 μm, respectively. When the voltage was 25 V, the width and depth of the microgroove were 475.9 μm and 119.8 μm, with less stray corrosion at the edges and a smoother bottom. The main reasons for this phenomenon are as follows: When the applied voltage is low, the passive film on the surface of the sample cannot be dissolved constantly, the amount of material removed is less, and the microgroove contour is shallower. 

**Figure 7:**
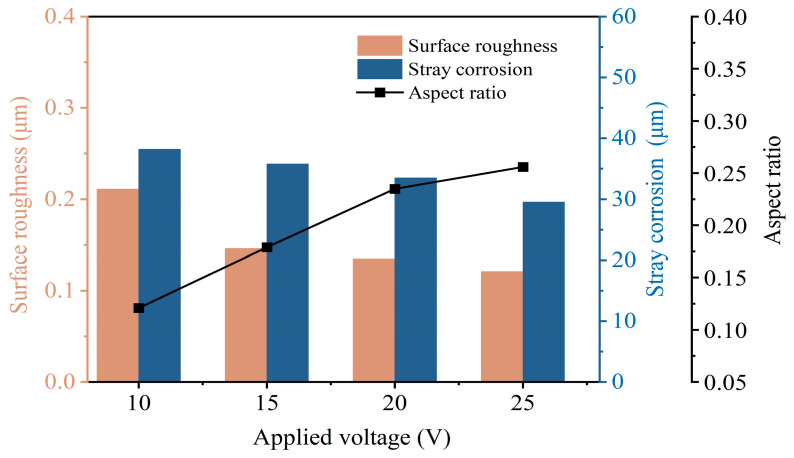
Influence of applied voltage on machining performance.

As shown in Figure 7, when the applied voltages were 10, 15, 20, and 25 V, the Ras were 0.211, 0.146, 0.135, and 0.129 μm, respectively. The surface roughness of Vit1 produced in the NaNO_3_ electrolyte is mainly dependent on the voltage applied to the machined surface, with smoother surfaces obtained at higher applied voltages. The surface roughness and stray corrosion show a decreasing trend. However, the aspect ratio gradually increases. With an increase in applied voltage from 10 V to 25 V, the aspect ratio increased from 0.121 to 0.252, the stray corrosion was reduced from 38.21 μm to 29.54 μm, and the surface roughness was changed from 0.211 μm to 0.129 μm. According to the results of studies, higher applied voltages can decrease stray corrosion and surface roughness. Consequently, 25 V was used as the applied voltage for jet-ECM of microgrooves.

#### 3.2.2. Effects of Nozzle Travel Rate

Unlike stationary microdimples, microgrooves remove material through relative motion between the tool electrode and the workpiece. The tool electrode moves at a relatively fast rate, leading to significant changes in the electric field. However, the relative motion between the tool electrode and the workpiece is too slow and there may be excessive accumulations of products which cannot be discharged in time. Therefore, the nozzle travel rate has a significant impact on the surface quality of the machining. Jet-ECM was performed with the following machining parameters: a NaNO_3_ electrolyte concentration of 10 wt%, an initial gap of 200 μm, an applied voltage of 25 V, and a nozzle travel rate of 100 to 400 μm/s.

**Figure 8:**
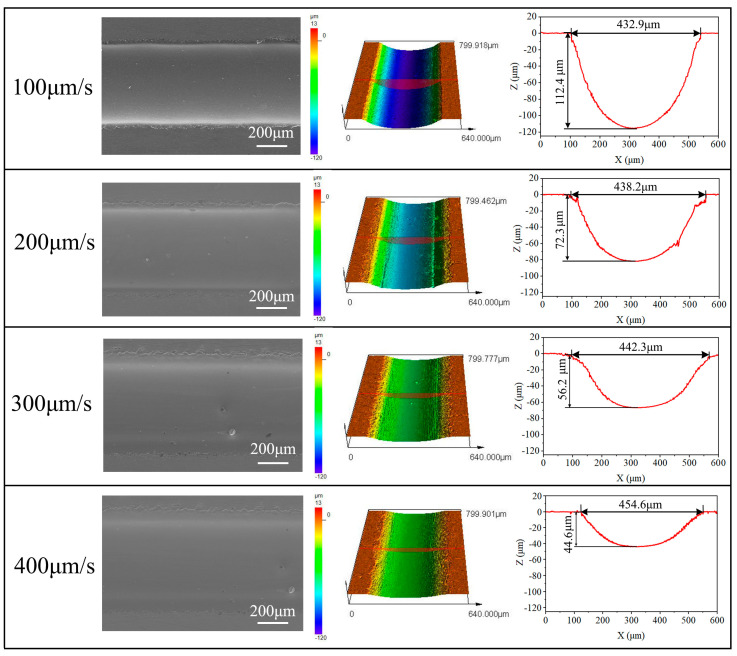
Morphology and profiles of microgrooves at different nozzle travel rates.

Figure 8 exhibits the machining parameters of the grooves obtained using different nozzle travel rates (100, 200, 300, and 400 μm/s). At a nozzle travel rate of 100 μm/s, the bottoms of the microgrooves exhibited relatively smooth surfaces, characterized by distinct boundaries and minimal stray corrosion. At this point, the width and depth of the microgrooves were 432.9 μm and 112.4 μm, respectively. With the increase in the nozzle travel rate, the depth of the microgrooves had a tendency to decrease gradually, and the edge contours became less clear. When the nozzle moving speed reached 400 μm/s, the fast movement caused a drastic change in the current density distribution, resulting in minimal anode material removal. Consequently, the width and depth of the microgrooves were only 454.6 μm and 44.6 μm, respectively.

**Figure 9:**
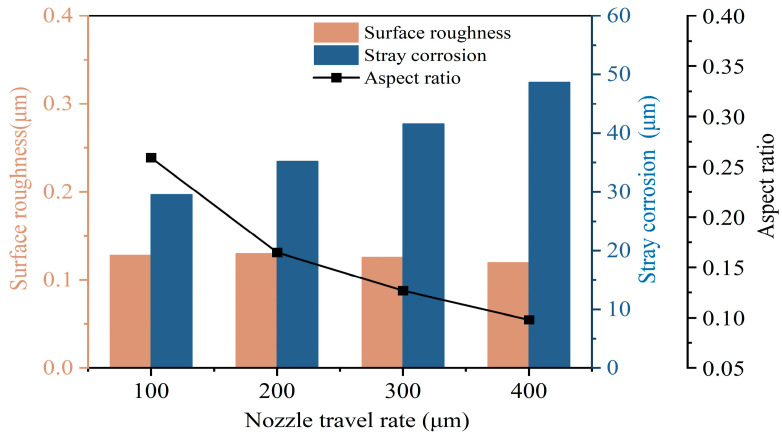
Influence of nozzle travel rate on machining performance.

The main machining quality indicators (aspect ratio, stray corrosion, and surface roughness) measured at different nozzle travel rates are shown in Figure 9. The aspect ratio decreases from 0.259 to 0.098 as the nozzle travel rate increases. Additionally, the stray corrosion range increases from 29.54 μm to 48.70 μm. Surface roughness tends to decrease from 0.128 μm to 0.120 μm. Due to the high-speed scouring of the electrolyte, the product can easily be eliminated, resulting in a smoother surface. Considering both machining efficiency and precision, better machining results are achieved at a nozzle travel rate of 100 μm/s.

#### 3.2.3. The Dissolution Mechanism of the Zr-based MG in NaNO_3_

**Figure 10:**
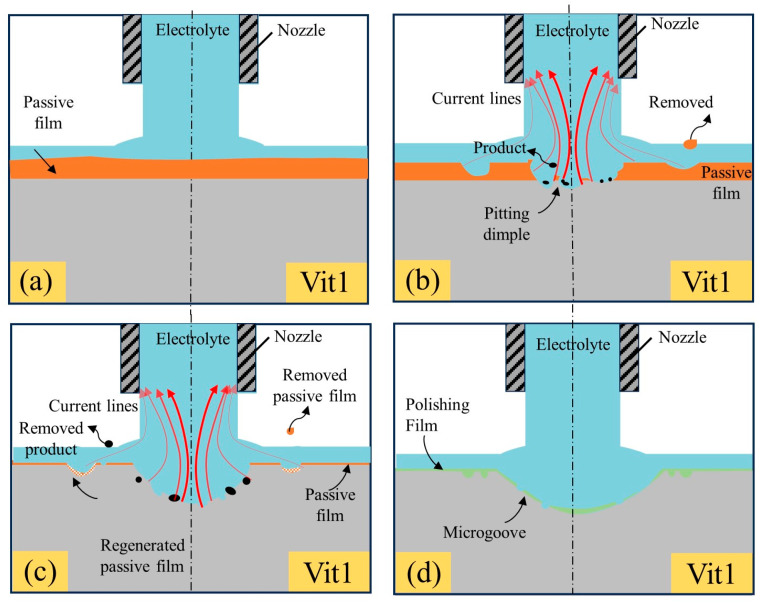
(**a**–**d**) Schematic of the dissolution mechanism of the Vit1 in 10 wt% NaNO_3_ solution.

Figure 10 illustrates a possible dissolution mechanism of the Vit1 in a 10 wt% NaNO_3_ solution. Based on the preceding discussion of polarization properties, the Vit1 exhibited behaviors of passivation, transpassivation, and re-passivation in NaNO_3_ solution.

As shown in Figure 10a, the passive film is generated on the surface of Vit1 and the thickness is uneven at the initial stage. As shown in Figure 10b, the scouring action of the fast-flowing electrolyte causes rupture of the film layer, leading to local dissolution and thinning of the passive film by jet electrochemical machining [37]. In the high-current-density region, pitting occurs on the surface of the anode material and dissolution of the material occurs. In the low-current-density region, partial rupture of the passive film occurs [36].

With an increase in both the number and dimensions of pits, the localized corrosion region expands as it intersects with neighboring minor pits, ultimately leading to the removal of the passive film covering the entire anodic surface and the stable dissolution of the anode material, as shown in Figure 10c. Due to the oxidation by ${\mathrm{NO}}_{3}^{-}$ ions in the passive electrolyte NaNO_3_. In the low-current-density region, the passivation film is regenerated, which is similar to that observed during reverse scanning for cyclic voltammetry [30]. As shown in Figure 10d, the anodic surface is covered with a layer of supersaturated nitrate film, which works as a polishing film and levels the anodic surface [38].

### 3.3. Fabrication of Precise and Smooth Microgroove Structures

Jet-ECM can selectively remove material with the multidimensional movement of the cathode nozzle [39]. This characteristic of jet-ECM renders it a highly flexible machining technique suitable for fabricating complex microstructures. A series of experiments were conducted using the following machining parameters: an applied voltage of 25 V, an initial gap of 200μm, a nozzle travel rate of 100 μm/s, and a NaNO_3_ electrolyte concentration of 10 wt%. 

**Figure 11:**
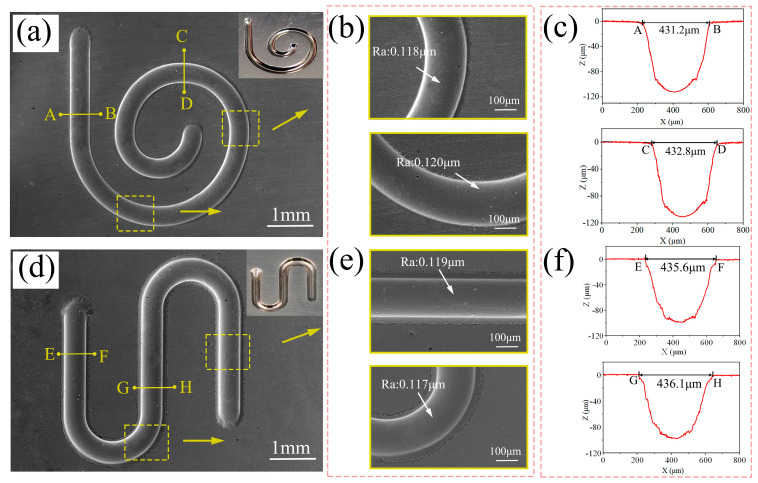
(**a**–**f**) Complex microstructures fabricated on the Vit1 by jet-ECM.

The complex microstructures fabricated on the Vit1 are depicted in Figure 11. As depicted in Figure 11a, both SEM and photographic images of the microhelical spiral are presented. The manufactured helical spiral structure exhibits clear edge contours and a smooth bottom. Figure 11b shows magnified SEM images of two regions with surface roughness values of 0.118 μm and 0.120 μm, respectively. Figure 11c displays the cross-sectional profiles of the groove bottoms in the two distinct regions. From measurements of the profiles in the cross-sections AB and CD, the results indicate microgroove widths of 431.2 µm and 432.8 µm and depths of 102.3 µm and 102.4 µm.

In Figure 11d, both SEM and photographic images of the micro-S structure are presented. Figure 11e displays magnified SEM images of two regions exhibiting surface roughness values of 0.119 μm and 0.117 μm. Figure 11f shows the cross-sectional profiles of the groove bottoms in the two distinct regions. Measurements of the profiles in the cross-sections EF and GH indicate microgroove widths of 435.6 µm and 436.1 µm, with depths of 101.1 µm and 99.8 µm. Additionally, the widths, depths, and surface roughness values of the microstructures were calculated to be 433.7 ± 2.4 µm, 101.4 ± 1.6 µm, and 0.118 ± 0.002 µm, which indicates the consistency of the contour dimensions of the fabricated microstructures. Therefore, jet electrochemical machining can manufacture precise and smooth microgroove structures on the Zr-based MG by using sodium nitrate solution.

## 4. Discussion

In this study, the electrochemical properties of Zr-based MG in NaNO_3_ solution and the feasibility of manufacturing precise and smooth microgrooves using jet-ECM were investigated. The main conclusions are summarized as follows:

1. Electrochemical characteristics indicate that Zr-based MG exhibited passive, trans-passive, and re-passive performances. An applied voltage higher than the transpassivation potential is required for jet electrochemical machining of Zr-based MG.
2. Jet-ECM can attain precise and smooth microgroove structures on the Zr-based metallic glass using a sodium nitrate electrolyte, with processing parameters including an applied voltage of 25 V, a nozzle travel rate of 100 μm/s, and a NaNO_3_ electrolyte concentration of 10 wt%.
3. High geometric dimensional consistency and low surface roughness microhelical and micro-S structures can be fabricated, and their widths and depths are 433.7 ± 2.4 µm and 101.4 ± 1.6 µm, respectively. Their surface roughness is 0.118 ± 0.002 µm, which represents a significant improvement over the structures obtained by non-aqueous-based ECM processes reported earlier.

## Figures and Tables

**Table 1:** Properties of the Zr-based MG.

Parameters	Value
Specific conductance (Ms/m)	0.52–0.53
Young’s modulus (GPa)	94.9
Poisson ratio	0.30
Hardness (HV)	568–619

**Table 2:** Processing conditions for jet-ECM.

Parameters	Value
Material	Zr_41.2_Ti_13.8_Cu_12.5_Ni_10.0_Be_22.5_
Tool electrode	SUS 304 nozzle
Inner diameter of nozzle	220 ± 2 μm
Outer diameter of nozzle	450 ± 3 μm
Electrolyte composition	10 wt% NaNO_3_
Machining gap (μm)	200
Electrolyte pressure (MPa)	1
Machining voltage (V)	10, 15, 20, 25,
Nozzle travel rate (μm/s)	100, 200, 300, 400
Temperature of electrolyte (°C)	25 ± 5

**Table 3:** Fitting results of the Vit1.

Potential (V)	R_1_ (Ω cm^−2^)	Q_1_ × 10^−6^ (s^n^Ω^−1^ cm^−2^)	n_1_	R_2_ (Ω cm^−2^)	R_3_ (Ω cm^−2^)	L_1_ (H cm^−2^)
0.1	16.36	17.96	0.91	182,770	-	-
1	8.49	8.13	0.93	78,989	-	-
2.1	6.55	10.21	0.93	44.18	15.06	0.064

## Data Availability

Data are contained within the article.
